# Trunk orthosis with joints providing resistive force improves dynamic sagittal alignment in postoperative patients with lumbar spinal stenosis

**DOI:** 10.1038/s41598-023-46209-6

**Published:** 2023-11-24

**Authors:** Tatsuya Igawa, Ken Ishii, Junji Katsuhira, Akifumi Suzuki, Hideto Ui, Ryunosuke Urata, Norihiro Isogai, Yutaka Sasao, Ko Matsudaira, Haruki Funao

**Affiliations:** 1 Department of Orthopaedic Surgery, School of Medicine, International University of Health and Welfare, 852 Hatakeda, Narita, Chiba 286-8520 Japan; 2 Department of Rehabilitation, International University of Health and Welfare Mita Hospital, 1-4-3, Mita, Minato-ku, Tokyo 108-8329 Japan; 3 Department of Physical Therapy, School of Health Science, International University of Health and Welfare, 2600-1, Kitakanemaru, Ohtawara, Tochigi 323-8501 Japan; 4 Department of Orthopaedic Surgery, Keio University School of Medicine, 35 Shinanomachi, Shinjuku-ku, Tokyo 160-8582 Japan; 5 Department of Medical Research and Management for Musculoskeletal Pain, 22nd Century Medical and Research Center, The University of Tokyo, 7-3-1, Hongo, Bunkyo-ku, Tokyo 113-8655 Japan; 6 Department of Human Environment Design, Toyo University, 1-7-11, Akabanedai, Kitaku, Tokyo 115-053 Japan; 7 Department of Pain Medicine, Fukushima Medical University School of Medicine, 1 Hikarigaoka, Fukushima, Fukushima 960-1295 Japan

**Keywords:** Medical research, Health care, Rehabilitation, Surgery

## Abstract

This study aimed to determine whether a trunk orthosis with joints providing resistive force (TORF) modifies sagittal malalignment during level walking in patients with lumbar spinal stenosis (LSS). Fifteen patients, 6 months after undergoing surgery for LSS, performed level walking at a self-selected speed while wearing a TORF. Dynamic sagittal alignment, including sagittal vertical axis, lumbar lordosis, and pelvic tilt, and spatiotemporal data as well as lower limb kinematic and kinetic data were recorded using a three-dimensional motion analysis system and six force plates. Statistical analysis was performed to compare these data with and without the TORF, respectively. Compared to the condition without the TORF, the use of the TORF significantly decreased positive sagittal vertical axis (p < 0.05) and increased the lumbar lordosis and pelvic tilt (p < 0.05). Peak hip flexion angle and extension moment during loading response (LR) significantly increased (p < 0.05), and peak hip extension angle and flexion moment during PS statistically decreased (p < 0.05). There was no difference in spatiotemporal data between the two conditions. Our findings suggest that TORF may modify the dynamic sagittal global alignment and lower limb kinematic and kinetics in postoperative LSS patients during level walking.

## Introduction

Lumbar spinal stenosis (LSS) is a disease with high morbidity, and it affects approximately 10% of the Japanese population aged > 60 years^1^. A typical symptom of this disease is severe neurological leg pain, numbness, and low back pain (LBP). Patients with LSS tend to walk with their trunk leaning forward because symptoms are relieved by leaning forward^2^. Neurological symptoms are alleviated by surgery including spinal canal decompression and/or spinal fusion^3, 4^. Surgical treatment is particularly effective in patients with severe stenosis and improves walking ability with the recent advances in minimally invasive surgery^5, 6^. However, since it has been reported that walking in a forward-bending posture continues even after decompression surgery^7^, it can be predicted that the preoperative posture for assisting nerve compression in forward bending has been learned and remains.

Global sagittal alignment is important for the outcomes of lumbar degenerative diseases^8–10^. Positive sagittal vertical axis (SVA) has a correlation with LBP, poor clinical outcomes, and health-related quality of life^9–11^. There has been a consensus that restoration of sagittal spinopelvic alignment, particularly normalization of the SVA, is indispensable for reasonable clinical outcomes^9^. A postoperative spinal orthosis therapy has been thought to immobilize segmental motion, enhance the fusion rate and reduce pain^12, 13^. However, it is unclear whether the use of orthosis contributes to the improvement of malalignment after lumbar surgery. Katsuhira et al. developed a trunk orthosis with joints providing resistive force (TORF; Trunk Solution Co., Ltd, Tokyo, Japan)^14^. They reported that TORF could enhance the forward tilt of the pelvis and upright posture of the trunk while decreasing low back extension moment with decrease of the low back flexion moment during level walking^14^. Thus, we predicted that the TORF will modify malalignment for postoperative patients with LSS, especially to improve SVA. Furthermore, we hypothesized that changes in sagittal alignment would also affect the kinematics of the lower limbs during walking. To our best knowledge, there are no reports of TORF improving dynamic sagittal alignment after lumbar surgery. The purpose of this study was to preliminarily assess whether TORF is useful in improving postoperative trunk anteversion gait by assessing dynamic sagittal alignment in patients with LSS.

## Results

The average age of the patients participating in this study was 65.9 years, and approximately 65% of the patients were male. In lumbar surgery, 60% of the patients underwent decompression alone and 40% underwent fusion surgery. Of all participants, 7 (47%) had SVA of > 40 mm and 10 patients (67%) had PI-LL > 10.

**Figure 1:**
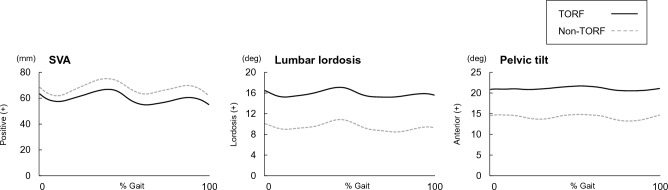
Comparison of SVA and the lumbar and the pelvic angle between the TORF and non-TORF conditions. The black-solid line represents the TORF condition, and the gray-dotted line indicates the non-TORF condition. *TORF* trunk orthosis with joints providing resistive force.

**Figure 2:**
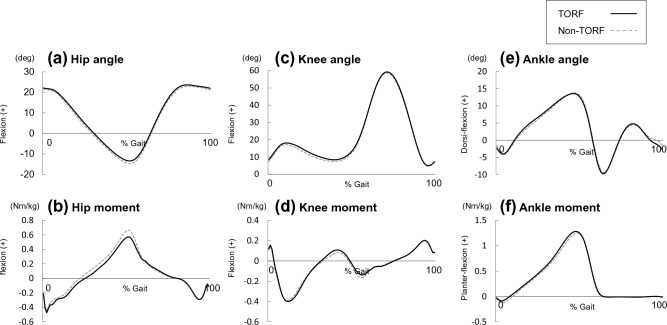
Comparison of the lower limb kinematics and kinetics between the TORF and non-TORF conditions. (**a**) Hip joint kinematics, (**b**) hip joint kinetics, (**c**) knee joint kinematics, (**d**) knee joint kinetics, (**e**) ankle joint kinematics, and (**f**) ankle joint kinetics. The black-solid line represents the TORF condition, and the gray-dotted line indicates the non-TORF condition. *TORF* trunk orthosis with joints providing resistive force.

Some kinematic parameters showed significant changes under the TORF condition. The dynamic SVA (dSVA) significantly decreased in TORF condition compared to the condition without the TORF (p < 0.05) (Table 1, Fig. 1a). The dynamic lumbar lordosis (dLL), calculated as the relative angle of the thorax to the pelvic segment, and dynamic pelvic tilt (dPT) significantly increased (p < 0.01) (Table 1, Fig. 1b,c). Subgroup analysis revealed that both the decompression group and the fusion group showed significant improvement in dynamic sagittal alignment (Table 2). No change was observed in spatiotemporal parameters (Table 3). Further, significant differences were found in the hip and ankle kinematics and kinetics (Table 4, Fig. 2). Peak hip flexion angle and extension moment during LR significantly increased under the condition with trunk orthosis compared with the condition without trunk orthosis (p < 0.05). Peak hip extension angle and flexion moment during PS decreased significantly (p < 0.05). Peak ankle plantar-flexion moment during mid-stance and terminal stance (MTS) and pre-swing (PS) significantly increased in TORF condition compared to the condition without the TORF (p < 0.05), while there were no statistically significant differences in terms of peak dorsi-flexion angle during MTS and PS.

**Table 1:** Comparison of dynamic sagittal alignment between walking with and without trunk orthotics.

	Normal gait		TORF gait		*p* value
	Mean	(SD)	Mean	(SD)	
Maximum dSVA (positive+)					
During LR (mm)	69	(28)	64	(28)	0.062
During MTS (mm)	77	(26)	69	(28)	**0.014**
During PS (mm)	70	(28)	61	(31)	**0.009**
During SW (mm)	73	(27)	64	(28)	**0.002**
Maximum dLL (lordosis+)					
During LR (deg)	15.2	(4.5)	21.7	(5.8)	** < 0.001**
During MTS (deg)	15.3	(4.5)	22.0	(5.7)	** < 0.001**
During PS (deg)	15.3	(4.5)	21.9	(5.9)	** < 0.001**
During SW (deg)	15.5	(4.3)	21.9	(5.9)	** < 0.001**
Maximum dPT (anterior+)					
During LR (deg)	9.1	(3.9)	15.2	(3.6)	** < 0.001**
During MTS (deg)	8.7	(3.7)	14.9	(3.5)	** < 0.001**
During PS (deg)	8.7	(3.7)	14.9	(3.7)	** < 0.001**
During SW (deg)	8.2	(3.5)	14.6	(3.7)	** < 0.001**

*dSVA* dynamic sagittal vertical axis, *dLL* dynamic lumbar lordosis, *dPT* dynamic pelvic tilt, *LR* loading response, *MTS* mid-stance and terminal stance, *PS* pre-swing, *TORF* trunk orthosis with joints providing resistive force.

Bold figures indicate statistically significant p < 0.05. Unit is degree.

**Table 2:** Subgroup analysis of dynamic sagittal alignment between walking with and without trunk orthotics.

	Decompression surgery				*p* value	Fusion surgery				*p* value
	Normal gait		TORF gait			Normal gait		TORF gait		
	Mean	(SD)	Mean	(SD)		Mean	(SD)	Mean	(SD)	
Maximum dSVA (positive+)										
During LR (mm)	73	(24)	72	(21)	0.297	59	(31)	51	(34)	0.118
During MTS (mm)	81	(23)	76	(20)	0.277	72	(31)	58	(34)	**0.015**
During PS (mm)	74	(26)	70	(20)	0.235	64	(30)	48	(39)	**0.015**
During SW (mm)	79	(25)	72	(20)	**0.042**	64	(27)	53	(34)	**0.019**
Maximum dLL (lordosis+)										
During LR (deg)	15.6	(5.2)	20.9	(5.4)	** < 0.001**	14.5	(3.4)	23.0	(6.5)	** < 0.001**
During MTS (deg)	15.8	(5.0)	22.1	(5.2)	** < 0.001**	14.7	(3.6)	23.3	(6.5)	** < 0.001**
During PS (deg)	15.8	(5.0)	21.0	(5.3)	** < 0.001**	14.6	(3.6)	23.2	(6.7)	** < 0.001**
During SW (deg)	16.0	(4.9)	21.0	(5.4)	** < 0.001**	14.8	(3.5)	23.3	(6.6)	** < 0.001**
Maximum dPT (anterior+)										
During LR (deg)	9.3	(3.6)	14.4	(3.1)	** < 0.001**	8.8	(4.4)	16.5	(4.0)	** < 0.001**
During MTS (deg)	9.0	(3.5)	14.1	(3.1)	** < 0.001**	8.3	(4.3)	16.2	(3.9)	** < 0.001**
During PS (deg)	8.9	(3.5)	14.0	(3.2)	** < 0.001**	8.4	(4.2)	16.2	(4.2)	** < 0.001**
During SW (deg)	8.5	(3.3)	13.7	(3.1)	** < 0.001**	7.8	(4.0)	16.0	(4.2)	** < 0.001**

*dSVA* dynamic sagittal vertical axis, *dLL* dynamic lumbar lordosis, *dPT* dynamic pelvic tilt, *LR* loading response, *MTS* mid-stance and terminal stance, *PS* pre-swing, *TORF* trunk orthosis with joints providing resistive force.

Bold figures indicate statistically significant p < 0.05. Unit is degree.

**Table 3:** Comparison of temporal and spatial parameters between normal gait and TORF gait.

	Normal gait		TORF gait		*p* value
	Mean	(SD)	Mean	(SD)	
Speed (m/s)	1.01	(0.11)	1.00	(0.13)	0.733
Stride length (m)	1.09	(0.11)	1.07	(0.11)	0.363
Cycle time (s)	1.08	(0.08)	1.08	(0.10)	0.514

*TORF* trunk orthosis with joints providing resistive force.

**Table 4:** Comparison of kinetic and kinematic lower limb parameters between conditions with and without trunk orthoses.

	Normal gait		TORF gait		*p* value
	Mean	(SD)	Mean	(SD)	
Hip joint					
Peak flexion angle during LR (deg)	21.8	(2.7)	22.4	(2.7)	**0.039**
Peak extension moment during LR (Nm/kg)	0.52	(0.19)	0.56	(0.19)	**0.035**
Peak extension angle during PS (deg)	15.1	(3.4)	13.9	(3.6)	** < 0.001**
Peak flexion moment during PS (Nm/kg)	0.70	(0.18)	0.62	(0.17)	** < 0.001**
Knee joint					
Peak extension angle during MTS (deg)	6.6	(5.3)	7.6	(4.9)	** < 0.001**
Peak flexion angle during PS (deg)	42.4	(5.8)	43.8	(5.7)	**0.005**
Ankle joint					
Peak dorsi-flexion moment during LR (Nm/kg)	0.13	(0.04)	0.11	(0.06)	0.054
Peak dorsi-flexion angle during MTS (deg)	14.6	(4.3)	14.3	(4.4)	0.212
Peak planter-flexion moment during MTS (Nm/kg)	1.29	(0.25)	1.32	(0.27)	**0.046**
Peak dorsi-flexion angle during PS (deg)	13.1	(4.5)	12.9	(4.5)	0.434
Peak planter-flexion moment during PS (Nm/kg)	1.24	(0.24)	1.27	(0.25)	**0.043**

*LR* loading response, *MTS* mid-stance and terminal stance, *PS* pre-swing, *TORF* trunk orthosis with joints providing resistive force.

Bold figures indicate statistically significant p < 0.05.

## Discussion

We hypothesized that TORF effectively corrects sagittal imbalance and improves gait performance such as gait speed and stride length during level walking in postoperative LSS patients. Our findings partially support this hypothesis. Significant differences were observed in dSVA, dLL, and dPT between the conditions with and without TORF. This study is the first report in the world to compare the dynamic sagittal balance with or without trunk orthosis in postoperative patients with lumbar spine.

Sagittal balance is frequently used clinically in the evaluation of patients with spinal disease by assessing the vertical axis of the sagittal plane. Static postural assessment using radiograph has some limitations and often does not reflect actual clinical results^15^. This is because there is a hidden sagittal imbalance in compensatory postural changes in the dynamic state^16^. It has been pointed out that traditional postural evaluation systems lack the dynamic aspects and hide some relevant information^17^. Measurement of gait parameters that complement static postural assessment is important for determining the objective therapeutic effect of surgery in LSS patients^18^. In this study, we evaluated changes in the dynamic sagittal balance and lower limb parameters due to the use of TORF in postoperative LSS patients using a 3-D gait analysis system.

Postoperative use of trunk orthotics has demonstrated effectiveness in pain relief, anxiety reduction, and promotion of healing; however, there exists conflciting opinions about its effects. Evidence regarding orthotic therapy after spinal surgery remains uncertain^19^. Furthermore, the effects of orthotic therapy on posture have never been investigated. Kuwahara et al.^20^ and Lames et al.^7^ analyzed trunk kinetics during walking in LSS patients who underwent decompression. They reported that the patients had a significantly more forward leaning trunk compared to healthy individuals of the same age. Sagittal spino-pelvic balance has recently gained attention because of its important role in maintaining the curvature of the entire spine. TORF has a posture correction effect and a facilitating effect of the abdominal muscles through resistive force applied on the chest^21^. It has been reported that the effect persists even after removal of TORF in stroke patients^22^. TORF might be used as one of the postoperative treatment options. This is because TORF that modify sagittal malalignment help increase the patient’s awareness of proper posture and reduces the risk of residual LBP in postoperative LSS patients. Improving sagittal alignment has many benefits for patients. Sagittal alignment has been reported to influence low back pain, quality of life, and risk of falling^9, 23, 24^. Furthermore, the results of subgroup analysis suggest that TORF may be effective in improving postoperative sagittal balance in patients without sacral-pelvic fixation or long-segment spinal fusion, regardless of fusion surgery. Based on past findings of TORF and the results of this study, we believe that it is effective to use TORF during walking training for rehabilitation in order to bring about the learning effect of improving posture in patients after LSS surgery. In addition, TORF has other potential applications because the clinical outcome of patients after vertebroplasty, especially for osteoporotic vertebral fractures^25, 26^, is greatly influenced by alignment after spinal surgery. In the future, it is necessary to verify the learning effect, dose, and long-term effect of this device.

There was no significant difference in walking performance including velocity, stride length, and cycle time between the conditions with or without TORF. It has been reported that the use of TORF improves gait performance in patients with stroke and total knee arthroplasties^22, 27^. The primary distinction between our study and previous studies is the subject’s medical condition. Previous studies have selected patients with lower limb dysfunction that significantly affects gait performance. It is inferred that the participants in our study already have sufficiently improved walking performance. In this study, patients participated 6 months after the surgery. At 3 weeks postoperatively, the patient had improved gait performance and good lower limb function^18^. Interestingly, there were also some significant differences between the two conditions in lower limb kinematics and kinetics, even though there was no change in the walking performance. After wearing the TORF, an increased hip flexion angle and extension moment during LR and a decreased hip extension angle and flexion moment during PS were observed. Lewis et al. have reported that walking in the anterior pelvic position increases the hip flexion angle and hip extension moment during LR and decreases the hip extension angle and flexion moment during PS compared to those in the posterior pelvic position^28^. From the results of analyzing the gait characteristics of spinal disease patients with posterior pelvic tilt, Igawa et al. showed that patients exhibit a reduced hip flexion angle and extension moment during LR, and an increased extension angle and flexion moment during PS^29^. The results of the present study is consistent with that of these previous studies. It is possible that the use of TORF facilitated the pelvis to lean forward and changed the kinematic and kinetic parameters of the hip joint. As the hip extension angle increases during walking, the anterior hip joint force increases^30^. Concurrent disease at both the hip and spine, as represented by hip-spine syndrome, is not infrequent in the older population^31^. It is suggested that postural modifications using TORF would reduce the forces on the anterior hip joint structures for patients after lumbar surgery.

This study has some limitations. First, this study is a descriptive study targeting postoperative patients without a control group. The causal relationship between posture improvement effects is unclear. Second, we have discussed the risk of residual LBP in LSS patients after surgery, focusing on the dSVA, dLL, and dPT during level walking. From the results of this study, it is not possible to mention the effect of improving residual LBP. This is because this study did not investigate changes in residual pain before and after wearing the TORF. The medium- to long-term effect of TORF cannot also be assessed from this study. Third, the subjects of this study were patients in a single institution. The results of this study are limited due to the possibility of selection bias, and joint research with other institutions is required in the future. Fourth, although this study emphasizes the importance of postural changes using the TORF, it is unclear if these changes are more beneficial than those provided by other orthotic devices. Finally, this study includes patients who have undergone different surgical procedures. There may be differences in posture depending on the surgical procedure.

In conclusion, we have demonstrated that the TORF can effectively correct the dynamic sagittal balance and the lower limb joints kinetics and kinematics in postoperative LSS patients. It is suggested that the TORF could be used as one of the treatment options for patients with LSS after surgery.

## Material and methods

### Features of the TORF

The features of the TORF have been described previously^22^. The TORF is mainly composed of three parts: pelvic support, upper support, and a joint with resistance. When attached, the pelvic support is fixed at the height of the superior anterior iliac spine and the superior posterior iliac spine, and the upper support is located anterior to the sternum and is adjusted approximately at the height of the ninth thoracic spine. Since the pelvic and upper support components are connected through the joint, when the trunk is tilted forward, the upper support pushes the chest by the tension of the spring. The resistance of the joint is exerted by the extension of the spring when the brace is attached, and the chest pressing force is exerted.

### Participants

Approval was granted by the Ethics Committee of the International University of Health and Welfare Mita Hospital (No. 5-16-26). Written informed consent was obtained from all subjects for publication of identifying information/images in an online open-access publication and participation in this study. All procedures performed in studies involving human participants were in accordance with the ethical standards of the institutional and/or national research committee, and with the 1964 Helsinki declaration and its later amendments or comparable ethical standards. We prospectively assessed gait characteristics for fifteen consecutive patients (mean age 65.9, range 49–80) approximately 6 months after undergoing decompression and/or spinal fusion for LSS from November 2018 to June 2019 at one hospital in Japan. The inclusion criterion was a clinical diagnosis of LSS by orthopedic surgeons. The diagnosis was physical assessment and confirmation of LSS by magnetic resonance imaging and X-ray computed tomography. The exclusion criterion was history of stroke, neuromuscular disease, cervical spondylotic myelopathy, severe hip and/or knee osteoarthritis. Patient demographics are shown in Table 5. Four senior surgeons performed all surgeries. Rehabilitation carried out during hospitalization after surgery was instructed by two of the authors (T. Igawa and A. Suzuki) to all patients, which included gait training and mild strengthening and stretching exercises of the leg and trunk muscles. Patients who underwent spinal fusion surgery used a rigid brace, and other patients used a soft brace. Both braces were used for 3 months after surgery and removed thereafter.

**Table 5:** Baseline demographic data of the participants.

Characteristic	n (%) or mean (SD)
Age, years	65.9 (7.9)
Sex (male)	15 (66.7)
BMI	25.7 (3.9)
Postoperative months	5.8 (1.3)
History surgery	
Decompression	9 (60.0)
L 4 laminectomy	1 (6.7)
L 4,5 laminectomy	4 (26.7)
L 2,3,4 laminectomy	2 (13.3)
L 3,4,5 laminectomy	1 (6.7)
L 2,3,4,5 laminectomy	1 (6.7)
Decompression + Fusion	6 (40.0)
L 1/2 TLIF	1 (6.7)
L 4/5 TLIF	2 (13.3)
L 4/5 PLIF	2 (13.3)
L 3/4/5 XLIF	1 (6.7)
Sagittal balance	
SVA (mm)	36.8 (31.3)
LL (deg)	47.9 (10.5)
PI (deg)	57.0 (5.8)
PT (deg)	21.9 (5.5)
SS (deg)	35.1 (6.3)
VAS	
Low back pain (mm)	13.2 (19.2)
Leg pain (mm)	13.3 (16.5)
ODI (%)	13.7 (15.0)
EQ5D-3L index	0.9 (0.2)

*BMI* body mass index, *TLIF* transforaminal lumbar interbody fusion, *XLIF* extreme lateral interbody fusion, *PLIF* posterior lumbar interbody fusion, *VAS* visual analog scale, *ODI* Oswestry disability index, *EQ5D* European quality of life-5 dimensions 3 Level.

### Outcome measures and data processing

**Figure 3:**
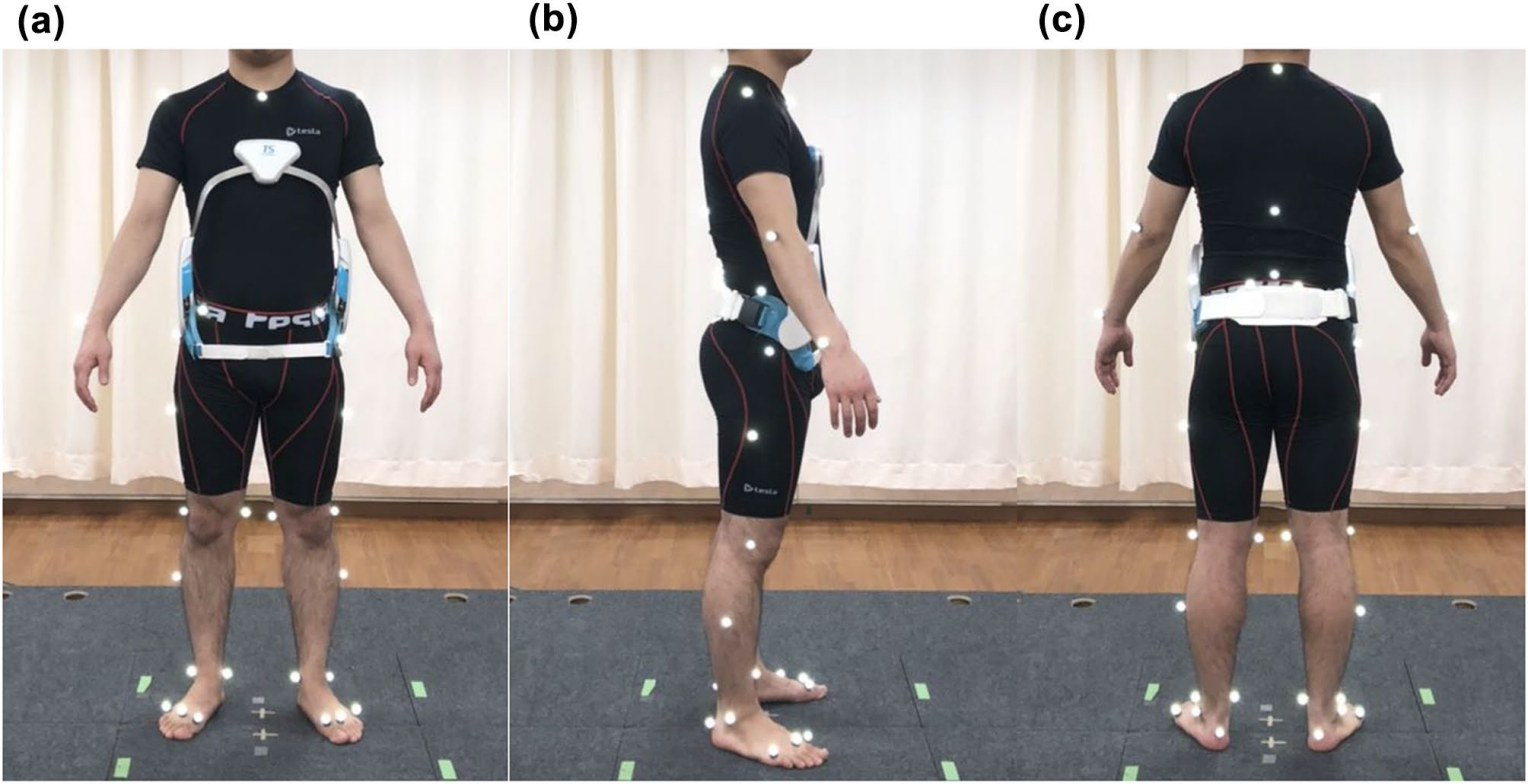
Forty-three reflective markers and trunk orthosis with joints providing resistive force. (**a**) Anterior view, (**b**) lateral view, and (**c**) posterior view.

Level walking was measured using a three-dimensional (3-D) motion analysis system consisting of 10 MX cameras (Vicon Motion Systems Ltd, Oxford, UK) and six force plates (AMTI, Watertown, MA, USA) at the patient’s selected speed with and without TORF. Kinematic and kinetic data were recorded at sample frequencies of 100 and 1000 Hz, respectively. To construct anatomical coordinate systems for each body segment, 43 reflective markers with a diameter of 14 mm were used as anatomical markers. These were attached to the landmarks, as per the Helen Hayes and Plug-in-gait Marker protocol (Fig. 3)^2, 32^. The subjects practiced walking for 5 min at self-selected speed in the laboratory settings before starting two different conditions. Subjects were provided sufficient rest time before conducting the measurements. To eliminate the residual effect of the TORF, all subjects conducted non-TORF condition and then TORF condition. Gait measurement was performed twice at self-selected speed for each condition.

Joint kinematics and kinetics were analyzed using Visual3D 3.6 analytical software (C-motion, Germantown, MD, USA). The recorded data were low-pass filtered using a second-order recursive Butterworth filter with respective cutoff frequencies of 6 and 18 Hz. Sagittal dynamic spinal alignments during gait were defined as dSVA, dLL, and dPT which were the primary outcome in the present study. The dSVA was calculated by the sagittal distance between the reflective marker on the spinous process of C7 and the midpoint coordinates of the posterior superior iliac spine markers^33^. The dLL was calculated by the relative angle between the thoracic and pelvic segments, referring to the method of Tojima et al.^34^. The thoracic segment was defined using the left and right iliac crest and shoulder markers. The seventh cervical vertebra, suprasternal notch, xiphoid process, and 10th thoracic vertebra markers were used as a cluster marker. The pelvic segment was defined using the CODA method from the anterior and posterior superior iliac spine markers. In the analysis, segments were regarded as rigid, and internal joint moments were calculated using a link segment model where segments were connected at nodal points. Thoracic tilt and dPT were calculated based on absolute angles of the thoracic and pelvic segments with reference to the method of Lamas et al.^7^.

Spatiotemporal data and kinetic and kinematic parameters of lower limb were set as the secondary outcome. Peak values for kinetic and kinematic parameters at the time of (1) LR, (2) MTS, and (3) PS were extracted for analysis. We defined these phases using the vertical component of the ground reaction force (cut off value; 20N). Joint moments were normalized by body mass. The average value of the data of two gait cycles on both limbs, or four gait cycles was used statistical analysis.

### Statistical analysis

Power analysis was performed using G*Power 3.1 (Heinrich Heine University, Düsseldorf, Germany). The sample size (15 patients) was determined to be able to detect a difference between with and without TORF for the change in pelvis angle^35^, assuming an effect size of 0.7, a type I error probability of 5% and a type II error probability of 30% (i.e. power of 70%).

To identify differences in kinematic and kinetic parameters depending on the use of TORF, the Wilcoxon signed-rank test was used for data that were not normally distributed, and the paired t-test was used for data that were normally distributed using the Shapiro–Wilk test. Subgroup analyzes were performed by classifying patients with and without fusion surgery. The effect of TORF on dynamic sagittal alignment was investigated respectively. A p-value of 0.05 was considered statistically significant. All statistical analyses were performed using SPSS for Windows version 25 software (IBM Corp., Armonk, NY, USA).

## Data Availability

All data generated or analyzed during this study are included in this published article.
